# The usefulness of quantifying intraepidermal nerve fibers density in the diagnostic of diabetic peripheral neuropathy: a cross-sectional study

**DOI:** 10.1186/s13098-016-0146-4

**Published:** 2016-04-11

**Authors:** Bogdan Timar, Simona Popescu, Romulus Timar, Flavia Baderca, Bogdan Duica, Mihaela Vlad, Codrina Levai, Bogdan Balinisteanu, Mihaela Simu

**Affiliations:** Department III – Functional Sciences, “Victor Babes” University of Medicine and Pharmacy, 2 Eftimie Murgu, 300041 Timisoara, Romania; Department VII – Internal Medicine, “Victor Babes” University of Medicine and Pharmacy, 2 Eftimie Murgu, 300041 Timisoara, Romania; Department II – Microscopic Morphology, “Victor Babes” University of Medicine and Pharmacy, Timisoara, Romania; Department X – Surgery, “Victor Babes” University of Medicine and Pharmacy, Timisoara, Romania; Legal Department, “Victor Babes” University of Medicine and Pharmacy, Timisoara, Romania; Department VIII – Neurosciences, “Victor Babes” University of Medicine and Pharmacy, Timisoara, Romania

**Keywords:** Skin biopsy, Intraepidermal nerve fibers density, Diabetic peripheral neuropathy

## Abstract

**Background:**

Distal symmetric polyneuropathy (DSPN) is the most common complication of type 2 diabetes mellitus (T2DM) and the most common form of peripheral neuropathy. DSPN increases the risk of foot ulceration up to seven-fold, and is a significant risk factor in more than 60 % of the amputations of the lower limbs in patients with T2DM. The aims of our study were to evaluate the difference in the density of intraepidermal nerve fibers (IENF) in patients with respectively without DSPN, to evaluate the strength of the relationship between the symptomatology of the DSPN and IENF density and to define a cutoff value of the IENF density for the diagnosis of DSPN.

**Methods:**

We enrolled, according to a consecutive, population-based method, 36 patients with T2DM admitted in our Clinic. For all patients, we measured HbA1c, lipid profile, body mass index and we assessed the presence and severity of DSPN using the evaluation of clinical symptoms, nerve conduction velocity and IENF density quantification.

**Results:**

The presence of neuropathy was significantly associated with a decreased density of IENF for both the proximal (11.6 vs. 14.9 fibers/mm; p = 0.014) and the distal biopsies (7.2 vs. 8.6 fibers/mm; p = 0.020). The optimal threshold value of IENF density (the point with the maximum sum of specificity and sensitivity), according to our model, was 10.1 fibers/mm.

**Conclusions:**

Skin biopsy followed by IENF density quantification is a valid, reliable tool for the diagnosis of DSPN.

## Background

It is well known that during its clinical course, diabetes may be accompanied by multiple other complications: microvascular and macrovascular [1], in the same time, diabetes being the most common cause of peripheral neuropathy in the world. Approximately half of patients with diabetes have neuropathy and among all patients having different neuropathies half of them are diagnosed with diabetes [2]. Distal symmetric polyneuropathy (DSPN) is the most common neuropathic complication of diabetes and the most common form of peripheral neuropathy worldwide. Depending on how neuropathy is defined, at least 50 % of patients with diabetes will develop DSPN, and up to 20 % already have DSPN at the time of diabetes diagnosis [3–6].

The diabetic neuropathies are a major cause of morbidity and mortality [7]. Approximately 20 % of patients with DSPN experience severe pain, DSPN being a major cause of disability and reduced quality of life [8, 9]. The most feared complication of DSPN is foot ulceration, which may eventually lead to foot or limb amputations: DSPN increases the risk of ulceration sevenfold and contributes to over 60 % of lower extremity amputation in patients with diabetes [10]. Early diagnosis of neuropathy might provide a mean to identify patients at high risk for lower-limb complications, and in the same time, allows early intervention and treatment leading thus to an improved prognosis for these patients.

Currently, for the diagnosis of diabetic neuropathy in clinical practice are used: the assessment of clinical symptomatology or the evaluation of nerve conduction velocity (NCV). These methods are cited to have advantages and disadvantages, ranging from the subjectivism of the patient or even of the examiner (i.e. the assessment of the symptomatology) to lack of specificity (NCV). The diagnostic method choice is as challenging for DSPN as in this case, especially in the early stages, only the small IENF are affected, so the changes might not be detected on NCV examinations. In this situation, the assessment of morphological changes of the small, intraepidermal nerve fibers (IENF) observed at the examination of the skin biopsy may be a more appropriate tool in the assessment of the presence and severity of DSPN.

The aims of our study were to evaluate the differences in the density of IENF in patients with respectively without DSPN on skin biopsies (both proximal and distal, as will be described in the method section). In addition, we evaluated the strength of the relationship between the symptomatology of the DSPN and IENF density, and defined a cutoff value of the IENF density for the diagnosis of DSPN.

## Methods

### Patients

We enrolled, according to a consecutive, population-based method, 36 patients with type 2 diabetes mellitus (T2DM) admitted in our Clinic. The study protocol was approved by the ethics committee of the Timisoara Emergency Clinical County Hospital, and the participants signed an informed consent at the time of recruitment.

For all patients, we measured the Hemoglobin A1c (HbA1c), lipid profile, body mass index (BMI) and we assessed the presence and severity of DSPN using the evaluation of signs and symptoms (using the Michigan Neuropathy Screening Instrument), NCV and IENF density quantification.

Michigan Neuropathy Screening Instrument (MNSI) is a score instrument designed for diagnosis and severity evaluation of DSPN which contains a patient’s symptoms questionnaire and clinical assessment tool. We considered positive for overt neuropathy the presence of one out of the following criteria: a global score ≥9.5 respectively questionnaire score ≥7 or clinical score ≥2.5. A higher score is associated with more severe neuropathy.

Nerve conduction velocity is an objective method used to assess DSPN. A lower NCV is associated with more severe DSPN. For this method, flat, patch-style electrodes are placed on the skin at intervals over the nerve that is being examined. These electrodes give off low-intensity electrical impulses, which stimulate the nerve. This stimulation may feel like a slight electric shock, though it is not particularly painful. The impulses produced by this electrical current are viewed on a computer screen. This monitoring system allows us to determine how fast the impulses are traveling through the nerves. In this study, the nerve conduction velocity was measured in the sural nerve territory.

### Skin biopsy

Specimens were obtained under topical anesthesia with lidocaine using a sterile disposable 3 mm punch. For diagnostic purposes in patients with polyneuropathy one skin specimen was taken at the distal end of the leg—10 cm above the lateral malleolus—within the territory of the sural nerve. A further specimen was taken at the proximal thigh—20 cm below the iliac spine—to demonstrate either the length-dependent process typical of polyneuropathy or the absence of a length-dependent pattern, as observed in sensory neuropathies. The specimens were immediately fixed in 2 % periodate-lysine-paraformaldehyde (2 % PLP) for up to 24 h at −4 °C, and then kept in a cryoprotective solution overnight. After fixation, the tissue was serially cut (sections of 50 μm in thickness, perpendicular to the dermis) using a freezing microtome to obtain vertical sections, which were kept in cryoprotective solution at −20 °C until free-floating immunohistochemistry was performed. Protein gene product (PGP) 9.5 was the marker used to assess the density of IENF for diagnostic purposes in peripheral neuropathies. For PGP 9.5 immunostaining we performed heat-induced epitope retrieval with Novocastra Bond Epitope Retrieval Solution 1, a ready-to-use, pH 6.0 solution (Leica Biosystems, Newcastle Ltd, Newcastle UponTyne NE 12 8EW, UK) for 20 min. Endogenous peroxidase blocking was realised with 3 % hydrogen peroxide for 5 min. This step was followed by incubation with primary antibodies for 20 min: PGP 9.5 (Leica Biosystems, Newcastle Ltd, Newcastle UponTyne NE 12 8EW, UK) monoclonal mouse anti-human (clone 10.A1, RTU). The Bond Polymer Refine Detection System was used for visualisation 3,3 diamino-benzidine dyhidrochloride was applied as chromogen, for 10 min and hemotoxylin—5 min, for counterstain. The entire immunohistochemical procedure was performed with Leica Bond-Max (Leica Biosystems, Newcastle upon Tyne, UK) autostainer.

Using bright-field immunohistochemistry, individual PGP 9.5 positive IENF crossing the dermal–epidermal junction were counted at high magnification (X 40) in at least three non-consecutive sections. After measuring the length of the section surface the linear density of IENF can be calculated. The result was expressed as IENF per millimeter.

### Statistical analysis

Data were collected and analyzed using the SPSS v.17 software suite (SPSS Inc. Chicago, IL, USA) and are presented as mean ± standard deviations for continuous variables with Gaussian distribution, median and [interquartile range] for continuous variables without Gaussian distribution, or percentages for categorical variables. To assess the significance of the differences between groups, the Student t test (means, Gaussian populations), Mann–Whitney-U test (medians, non-Gaussian populations) and Chi square (proportions) tests were used. Continuous variable distributions were tested for normality using Shapiro–Wilk test, and for equality of variances using Levene’s test.

The strength of the association between two continuous variables from non-Gaussian populations was evaluated using Spearman’s correlation coefficient. Its statistically significance was assessed using t-score distribution’s test.

The quality of the diagnostic tests for continuous variables was evaluated using a receiver-operating characteristic (ROC) analysis. The optimum threshold for a positive diagnosis was calculated according to Youden’s method, where the selected value maximizes the sum of the sensitivity and specificity. As reference for comparison, we used the positive diagnosis of DSPN according to the MNSI criteria, as described previously.

In this study, p < 0.05 was considered the threshold for statistical significance.

## Results

### Studied group characteristics

The patients enrolled in our study (n = 36) had a median age of 65 years and a median diabetes duration of 9 years; 58.3 % (21) of them were men and 41.7 % (15) women. Our patients had an average HbA1c value of 8.2 % and an average BMI of 30.7 kg/m^2^. Regarding comorbidities and other diabetes complication, we observed that retinopathy was present in 36.1 % of the patients, chronic kidney disease in 30.6 % of them respectively hypertension in 61.2 %. The studied patient’s baseline characteristics are presented in Table 1.

**Table 1:** Patient’s baseline characteristics

Age (years)^a^	65 [14]
Male gender^b^	21 (58.3 %)
Diabetes duration (years)^a^	9 [7]
HbA1c (%)^c^	8.2 ± 1
Total cholesterol (mg/dL)^c^	206.9 ± 51.6
LDL cholesterol (mg/dL)^c^	123.8 ± 41.7
HDL cholesterol (mg/dL)^c^	45.2 ± 8.7
Triglycerides (mg/dL)^c^	189.6 ± 99.6
BMI (kg/m^2^)^c^	30.7 ± 3.5
Waist circumference (cm)^c^	106.2 ± 9.3
Retinopathy^b^	13 (36.1 %)
Chronic kidney disease^b^	11 (30.6 %)
Hypertension^b^	22 (61.2 %)

^a^Numerical, continuous variables without Gaussian distribution. Values are presented as median and [interquartile range]

^b^Dichotomous variables. Values are presented as numbers and (percentage from total)

^c^Numerical continuous variables with Gaussian distribution. Values are presented as mean ± standard deviation

### DSPN prevalence

We investigated the presence of DSPN using two approaches: the MNSI respectively the NCV. The median MNSI global score in our group was 6 points (median questionnaire = 4 respectively median clinical assessment 2 points). If the cutoffs values of 9.5 points (global score) or 7 points (questionnaire) or 2.5 points (clinical assessment) were used, the prevalence of DSPN in our cohort was 41.7 % (15 individuals).

The presence of DSPN was associated with a decreased NCV (38.7 ± 2.4 m/s) compared to patients without DSPN (42.4 ± 2.6 m/s), the differences being statistically significant (p < 0.001). In addition, the presence of DSPN was associated with higher age (69 vs. 62 years), HbA1c (8.5 vs. 8.0 %) and BMI (32.6 vs. 29.3 kg/m2) and longer diabetes duration (12 vs. 7 years). No significant associations were found between the presence of neuropathy and the total cholesterol, HDL cholesterol, LDL cholesterol respectively triglycerides values. The comparison of parameters between neuropathy present vs. absent groups is presented in Table 2.

**Table 2:** Comparison of studied parameters stratified by the presence of neuropathy

	Without DSPN (n = 21)	With DSPN (n = 15)	p
Age (years)^a^	62 [13]	69 [11]	0.027*
Diabetes duration (years)^a^	7 [5]	12 [7]	0.044*
HbA1c (%)^b^	7.9 ± 0.8	8.6 ± 1.2	0.043*
BMI (kg/m^2^)^b^	29.3 ± 3.6	32.6 ± 3.3	0.008*
Total cholesterol (mg/dL)^b^	216.1 ± 49.9	194.1 ± 52.8	0.211
LDL cholesterol (mg/dL)^b^	127.8 ± 43.7	118.2 ± 39.6	0.503
HDL cholesterol (mg/dL)^b^	46.1 ± 8.4	43.8 ± 9.3	0.432
Triglycerides (mg/dL)^b^	210.6 ± 106.7	160.3 ± 83.4	0.137

* Differences are statistically significant

^a^Numerical continuous variables without Gaussian distribution. Values are presented as median and [interquartile range]. p value was calculated using Mann–Whitney U test

^b^Numerical continuous variables with Gaussian distribution. Values are presented as mean ± standard deviation. p value was calculated using unpaired t-student test

### Association between IENF and DSPN

The presence of neuropathy was significantly associated with a decreased density of IENF both for the proximal and the distal biopsies (Table 3).

**Table 3:** Differences in IENF density in patients without and with DSPN

IENF density	Without DSPN	With DSPN	p
Proximal biopsy (fibers/mm)	14.9 ± 3.4	11.6 ± 4.1	0.014*
Distal biopsy (fibers/mm)	8.6 ± 1.6	7.2 ± 1.8	0.020*

Numerical continuous variables. Values are presented as mean ± standard deviation. *p* value was calculated using unpaired t-student test

* Differences are statistically significant

**Fig. 1:**
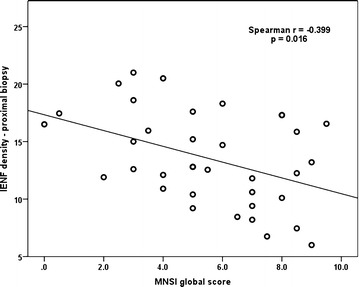
Correlation between MNSI global score and the density of IENF. MNSI: Michigan Neuropathy Screening Instrument; IENF: intraepidermal nerve fibers

We found a significant and moderate negative correlation between the density of IENF at the proximal biopsy and the MNSI score (Spearman’s r = −0.399; p = 0.016), which demonstrates that measuring the IENF density is a valid and reliable method for evaluating the DSPN. A lower density of IENF was associated in our study with more severe DSPN signs and symptoms, as evaluated using MNSI. The correlation between the MNSI global score and IENF density at the proximal biopsy is presented in Fig. 1.

**Fig. 2:**
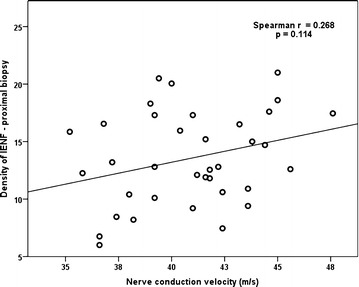
Correlation between NCV and the density of IENF at the proximal biopsy. NCVL: nerve conduction velocity; IENF: intraepidermal nerve fibers

The association between the density of IENF at the proximal biopsy and NCV was a positive and moderate one, however, in our study was only marginally significant one, most probable being caused by a β statistical error (Spearman’s r = 0.268; p = 0.114). The relationship indicates that a higher density of IENF is associated with a higher NCV, thus demonstrating again the usefulness of measuring the density of IENF in evaluating the DSPN. The correlation between the density of IENF at the proximal biopsy and NCV is presented in Fig. 2.

### Defining an IENF density threshold for the diagnosis of DSPN

To evaluate the prediction performance of DSPN diagnosis based on the density of IENF at the proximal biopsy site, we built a receiver-operating characteristics model, having as predictor the IENF density and as outcome the positive diagnosis of neuropathy based on the MNSI score criteria, as defined in “Methods” section. The density of IENF proved to be a valid discriminant for the diagnosis of DSPN, having an area under the receiver-operating curve (ROC) of 0.740, significantly different from a non-discriminant (area under ROC = 0.5), observing a p value of 0.007.

The optimal threshold value of IENF density (the point with the maximum sum of specificity and sensitivity), according to our model, was 10.1 fibers/mm, a cutoff point with a corresponding sensitivity of 0.47, specificity of 0.95, positive predictive value of 0.88 respectively negative predictive value of 0.71.

**Fig. 3:**
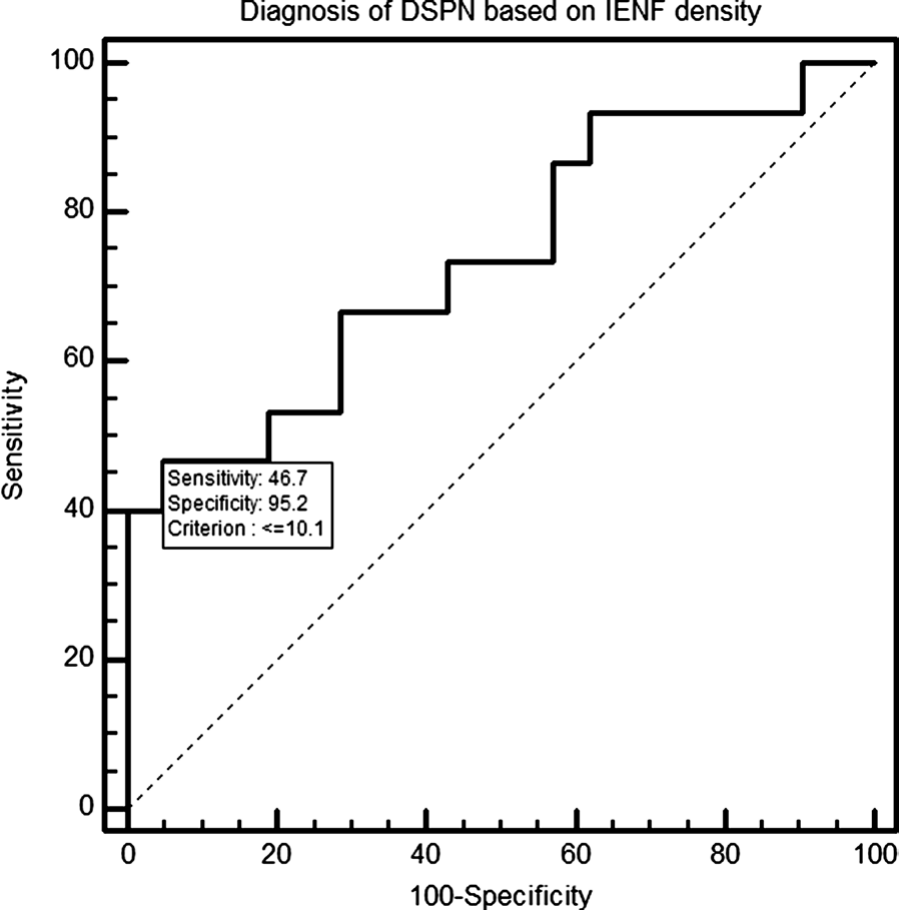
Receiver-operating characteristics analysis for IENF density. DSPN: diabetic sensitive polyneuropathy; IENF: intraepidermal nerve fibers

Based on our values, for diagnosing DSPN with a sensitivity of at least 0.95 a cutoff value of 18.6 fibers per millimeters should be chosen, respectively to diagnose DSPN with a specificity of at least 0.95 a cutoff value of 10.1 fibers/mm should be chosen. The ROC-curve analysis is presented in Fig. 3.

## Discussion

Small-nerve fiber dysfunction usually occurs early and is often present without objective signs or electrophysiological evidence of nerve damage [11], this is why the evaluation of morphological damages in the small, IENF may be an improved method for diagnosing and staging DSPN, especially in incipient and sub-clinical stages of progression.

The European Federation of the Neurological Societies and the Peripheral Nerve Society endorse intraepidermal nerve fiber quantification to confirm the clinical diagnosis of small fiber neuropathy with a strong (Level A) recommendation [12]. IEFN density is reverse correlated with both cold and heat detection thresholds [13]. IEFN density is significantly reduced in symptomatic patients with normal findings from nerve conduction studies and those with metabolic syndrome, impaired glucose tolerance, and impaired fasting glycemia, suggesting early damage of the small nerve fibers [14, 15]. Intraepidermal nerve fiber density is also reduced in painful neuropathy compared with that observed in painless neuropathy [16]. Diet and exercise intervention in impaired glucose tolerance patients leads to increased intraepidermal nerve fiber density [17]. These data suggest that intraepidermal nerve fiber loss is an early feature of the metabolic syndrome, prediabetes, and established diabetes, this loss progressing along with increases in neuropathic severity.

In 2005, a task force of the European Federation of Neurological Societies published guidelines on the use of skin biopsy in the diagnosis of peripheral neuropathies, in which the usefulness of the technique was established [12]. IENF density in the distal part of the leg has been found to be useful for confirming a diagnosis of peripheral neuropathy of various etiologies with specificity ranging from 95 [18] to 97 % [19], sensitivity ranging from 45 [18] to 80 % [19], a positive predictive value of 92 %, and a negative predictive value of 90 % [19]. Since 2005, other papers, focused on pure small-fiber neuropathy, have provided additional support for the important role of skin biopsy in diagnosis [20–23]. Small fiber neuropathy is frequently met in clinical practice, but can be misdiagnosed because of the absence of neurophysiological tests that can investigate small nerve fibers. In most studies, patients’ complaints—mainly burning feet—are taken as the gold standard against which the performance of skin biopsy is measured. Studies in patients with clinically suspected pure small-fiber neuropathy reported that skin biopsy had a sensitivity of 90 % and a specificity of 95 % [18, 24]. In idiopathic and secondary (diabetic, cytotoxic or amyloid) small fiber neuropathy, skin biopsy analysis showed a positive predictive value of 95 % and a negative predictive value of 91 [25].

To our knowledge, this study is the first one which is validating this method as a diagnosis tool for DN in the Romanian population of patients with T2DM, a population which may have a set of ethnical and socio-economical particularities in relation to other studied populations. More, in the context of the European Federation of Neurological Societies recommendation, that further studies are needed to improve and validate this method on more populations [12], we may agree that the impact of this types of studies, on new populations, is emphasized. Also, this results may be of a paramount importance when meta-analysis are to be performed in order to define a more accurate threshold for diagnosis.

One limitation of skin biopsy is that it cannot help in assessment of the etiology of neuropathy. The technique cannot replace nerve biopsy when neuropathological examination of mixed or large-fiber neuropathy is needed and when a vasculitis pathogenesis is suspected. Finally, despite high positive and negative predictive values in small-fiber neuropathy, normal IENF density cannot exclude a functional impairment of unmyelinated fibers.

It is of a paramount importance to be mentioned that an abnormal result obtained regarding the INEF density after skin biopsy, as described in this paper, should be followed by a detailed differential diagnosis since it is known that abnormal IENF density may be also observed in carpal or tarsal tunnel syndrome, in pure small fiber polyneuropathy and in various other types of neuropathies [26].

## Conclusions

Skin biopsy followed by IENF density quantification is a valid, reliable tool for the diagnosis of DSPN. It might provide further information and might be an improved diagnostic method compared to the traditional ones, especially in case of the small fibers neuropathy, a condition underdiagnosed in the past, a condition frequently associated with the presence of diabetes mellitus.

Skin biopsy samples can demonstrate the selective degeneration of somatic unmyelinated fibers that convey pain and thermal sensations and can also provide diagnostic information when there is little or no clinical evidence of neuropathy. The minimal invasiveness of the method makes it a useful tool not only in clinical practice, but also for monitoring the progression of neuropathy in trials of neuroprotective treatments. The procedure can be repeated close to the site of a previous biopsy, within the territory of the same sensory nerve thus allowing to investigate the progression of neuropathy and the effect of neuroprotective treatments.

Skin biopsy followed by the quantification of the IENF should be considered in patients with symptoms of small-fiber neuropathy when nerve conduction studies do not reveal abnormalities.

## Abbreviations

DSPN: distal symmetric polyneuropathy
IENF: intraepidermal nerve fibers
HbA1c: hemoglobin A1c
BMI: body mass index
MNSI: Michigan Neuropathy Screening Instrument
NCV: nerve conduction velocity
PGP: protein gene product
ROC: receiver-operating curve
